# Walking participation and health-related quality of life: an ecological analysis across 229 districts in Korea

**DOI:** 10.1186/s12889-026-27962-5

**Published:** 2026-06-11

**Authors:** Soo-jin Choi

**Affiliations:** Department of Education, Zhejiang University, Hangzhou, Zhejiang China

**Keywords:** Physical activity, Walking, Moderate-to-vigorous physical activity, Depression, Perceived stress, EQ-5D, Health-related quality of life, Ecological study, Korea

## Abstract

**Background:**

Walking is a widely accessible form of physical activity and has been consistently associated with improved health-related quality of life (HRQoL) at the individual level. However, less is known about how walking participation relates to HRQoL at the population level, particularly when mental health indicators are considered within an ecological framework.

**Methods:**

This study conducted an ecological analysis using publicly available aggregated data from 229 administrative districts in South Korea. District-level measures of walking participation, moderate-to-vigorous physical activity (MVPA), depressive symptoms, perceived stress, and HRQoL (EQ-5D) were analyzed. Pearson correlation analyses and multiple linear regression models were employed to examine associations among variables.

**Results:**

Walking participation was positively associated with HRQoL in the initial ecological models. However, these associations were attenuated and became non-significant after adjustment for district-level aging structure, while model explanatory power increased substantially. MVPA showed no significant association with HRQoL. Depressive symptoms were negatively associated with HRQoL, whereas perceived stress demonstrated a positive association at the district level. Walking participation was also positively associated with depressive symptoms and perceived stress at the district level, suggesting that district-level ecological associations may differ from individual-level relationships.

**Conclusions:**

At the ecological level, walking participation showed significant associations with HRQoL, although these associations were partly attenuated after adjustment for district-level aging structure. These findings highlight the importance of cautious interpretation when examining population-level health relationships using district-level aggregate data. Overall, the results suggest that walking participation may serve as a population-level marker associated with regional HRQoL patterns, rather than providing direct evidence that increasing walking alone would improve district-level HRQoL.

## Introduction

Walking is widely regarded as one of the simplest and most accessible forms of physical activity. It requires essentially no equipment and can be performed in almost any environment, which partly explains its popularity across age groups. A large body of research has shown that regular walking is linked to improvements in cardiovascular health, reductions in mortality risk, and better psychological well-being [1–4]. In public health scholarship, walking has increasingly been positioned as a core behavior that supports active aging and broad population health goals [5, 6]. Beyond its physiological benefits, walking contributes to social engagement, functional mobility, and independence, making it a particularly important behavior for population-level health promotion [7].

Health-related quality of life (HRQoL) is often evaluated using preference-based instruments such as the EQ-5D, which capture a range of physical, functional, and psychological domains [8, 9]. Because HRQoL synthesizes multiple dimensions of health status, it is frequently used in national surveillance systems and public health planning. Prior studies have generally reported that individuals who engage more frequently in walking tend to report higher HRQoL [1–4, 10–12]. However, the vast majority of this evidence derives from individual-level data, which are shaped by personal behaviors, motivations, and health conditions. These findings, therefore, do not necessarily translate directly to broader community or district-level patterns, where shared environmental exposures, policy contexts, and social structures may exert stronger influences [1].

Ecological analyses—those that examine relationships across aggregated populations—provide a complementary perspective to individual-level research by identifying regional patterns in health behaviors and outcomes [13–15]. Although individual-level studies have consistently demonstrated positive associations between walking and health-related quality of life (HRQoL), less is known about how these relationships manifest at the population level across administrative regions [1–4]. Examining district-level variations may offer useful descriptive insights for public health surveillance and regional health monitoring [13, 15]. In particular, ecological approaches can help identify geographic disparities in physical activity and quality of life that may not be fully captured through individual-level analyses alone [15]. Despite the growing use of regional health data in public health research, relatively few nationwide ecological studies have investigated district-level associations between walking participation and HRQoL.

Mental health indicators such as depressive symptoms and perceived stress are also closely related to physical activity and HRQoL [16–18]. While individual-level studies generally report that physical activity is associated with lower depression and stress, these relationships may appear differently at the ecological level because aggregate district-level indicators reflect both population composition and broader regional characteristics. For example, some districts may simultaneously report higher walking participation and higher perceived stress levels, although the present dataset does not allow these patterns to be attributed to specific contextual mechanisms such as urbanization, economic activity, or transportation infrastructure. Understanding these district-level patterns may therefore contribute to a more nuanced interpretation of population health relationships [19, 20].

Korea provides a particularly relevant setting for ecological public health research because its administrative districts exhibit substantial variation in urbanization, demographic structure, health behaviors, and health-related quality of life [21–23]. Rapid urban development, population aging, and regional inequalities have created distinct population health patterns across Korean districts. Using nationwide district-level statistics from 229 administrative districts (si–gun–gu), this study examines ecological associations among walking participation, mental health indicators, district-level aging structure, and HRQoL. The study is intended as a descriptive ecological analysis of district-level population patterns rather than as a direct test of contextual or environmental mechanisms. Because direct measures of socioeconomic conditions, urbanization, walkability, healthcare access, and other contextual characteristics were not included in the models, the findings should be interpreted cautiously and should not be viewed as evidence of specific contextual pathways.

## Materials and methods

### Study design

This study employed a cross-sectional ecological design using aggregated district-level health statistics from South Korea. The unit of analysis was 229 administrative districts (si–gun–gu), which represent the primary local administrative divisions used in Korean public health surveillance and regional policy planning. Ecological analyses were conducted to examine regional variations in walking participation, mental health indicators, and health-related quality of life (HRQoL) at the population level rather than at the individual level. This approach allows the identification of geographic patterns relevant to regional health monitoring and ecological interpretation of district-level population health indicators.

### Data source and regional units

All variables were derived from publicly available 2024 regional statistics provided through the Korean Statistical Information Service (KOSIS) and the Korea Community Health Survey (KCHS). The KCHS is a nationwide annual survey conducted by the Korea Disease Control and Prevention Agency (KDCA) to monitor regional health behaviors and health outcomes across administrative districts in South Korea. The survey uses a standardized multistage sampling design and trained interviewers to collect health-related information from community-dwelling adults aged 19 years and older.

The present study analyzed aggregated district-level statistics from 229 administrative districts (si–gun–gu). Higher-level aggregate rows (e.g., provincial subtotal categories) were excluded to ensure that all observations represented comparable municipal-level administrative units. These districts vary substantially in population size and demographic structure, making them appropriate units for ecological public health analysis.

All variables used in this study were publicly available in aggregated form, and no individual-level or identifiable data were accessed.

### Measures

This study included district-level indicators of physical activity, mental health, and health-related quality of life. All variables were obtained from the 2024 Korean Statistical Information Service (KOSIS) regional health datasets and were aggregated at the si–gun–gu (district) level.

#### Physical activity measures

Walking participation rate was defined as the percentage of adults aged 19 years and older in each district who reported walking for at least 30 min per day on five or more days per week. This indicator was derived from the Korea Community Health Survey and reflects regular walking participation at the population level.

Moderate-to-vigorous physical activity (MVPA) represented the percentage of adults aged 19 years and older meeting the nationally recommended physical activity guidelines in Korea, defined as engaging in moderate-intensity physical activity for at least 150 min per week, vigorous-intensity physical activity for at least 75 min per week, or an equivalent combination of both. Both indicators were obtained from district-level regional health statistics.

#### Mental health indicators (Depression and Stress)

Depressive symptoms were measured as the percentage of adults aged 19 years and older reporting depressive mood lasting for two consecutive weeks or longer during the past year. Perceived stress was defined as the percentage of adults aged 19 years and older reporting high levels of daily stress in their everyday lives. These indicators were obtained from district-level mental health items included in the Korea Community Health Survey and reported through KOSIS regional statistics.

#### Health-related quality of life (EQ-5D)

Health-related quality of life (HRQoL) was assessed using the EQ-5D index, a validated measure capturing five dimensions: mobility, self-care, usual activities, pain/discomfort, and anxiety/depression. District-level mean EQ-5D scores were used as the primary outcome variable, with values ranging from 0 (worst imaginable health state) to 1 (full health). In additional sensitivity analyses, district-level aging structure was included as an adjustment variable using the proportion of adults aged 65 years and older in each district. This variable was obtained from publicly available regional demographic statistics provided through KOSIS.

### Statistical analysis

Descriptive statistics were computed for all variables to assess regional variation across districts. Pearson correlation coefficients were used to examine bivariate associations among walking participation, MVPA, mental health indicators, and HRQoL.

Multiple linear regression models were estimated to examine the ecological associations between walking participation and HRQoL. Four models were constructed. Model 1 examined associations between physical activity indicators (walking participation and MVPA) and HRQoL. Model 2 examined associations between mental health indicators (depressive symptoms and perceived stress) and HRQoL. Model 3 (full model) simultaneously included walking participation, MVPA, depressive symptoms, and perceived stress. Model 4 additionally adjusted for district-level aging structure using the proportion of adults aged 65 years and older.

Additional regression analyses examined ecological associations between walking participation and district-level mental health indicators (depressive symptoms and perceived stress).All statistical analyses were conducted using Python (version 3.10) with the statsmodels library. Statistical significance was defined as *p* < 0.05.

To assess the robustness of the findings, additional sensitivity analyses were conducted using alternative model specifications, including adjustment for district-level aging structure. Variance inflation factor (VIF) values were examined to assess potential multicollinearity among predictors. All VIF values remained below conventional thresholds, indicating that multicollinearity was not a major concern in the analyses. Adjusted R² values were also examined to evaluate model explanatory power.

### Ethics approval and consent to participate

According to the ethics regulations of Zhejiang University, research using publicly available and fully de-identified secondary data is exempt from IRB review. Accordingly, this study received an official exemption from the Institutional Review Board of Zhejiang University. No individual-level or identifiable information was used.

## Results

### Descriptive statistics

Table 1 presents descriptive statistics for all district-level variables (*N* = 229). Walking participation showed a mean of 50.36% (SD = 11.13), while MVPA had a mean of 24.63% (SD = 6.55). Depressive symptoms averaged 6.62% (SD = 2.12), and perceived stress averaged 20.93% (SD = 3.53). The mean EQ-5D score was 0.938 (SD = 0.017), indicating relatively high HRQoL across districts with modest regional variation. The mean proportion of adults aged 65 years and older was 26.42% (SD = 9.31).

**Table 1:** Descriptive Statistics for All Variables (*N* = 229)

Variable	Mean	SD	Min	Max
Walking participation (%)	50.36	11.13	25.9	78.9
MVPA (%)	24.63	6.55	11.5	51.2
Depressive symptoms (%)	6.62	2.12	1.0	12.6
Perceived stress (%)	20.93	3.53	12.6	38.4
EQ-5D (HRQoL index)	0.938	0.017	0.889	0.975
Aged 65+ (%)	26.42	9.31	10.9	47.5

*MVPA* moderate-to-vigorous physical activity, *EQ-5D* EuroQol-5 dimensions

### Correlation analysis

Pearson correlations revealed several significant associations among the study variables (Table 2). Walking participation was positively correlated with EQ-5D (*r* = 0.27, *p* < 0.001), indicating that districts with higher walking participation tended to report higher HRQoL. Walking was also positively associated with depression (*r* = 0.19, *p* < 0.01) and stress (*r* = 0.32, *p* < 0.001), suggesting that district-level ecological associations between walking participation and mental health indicators may differ from individual-level relationships.

**Table 2:** Pearson Correlations Among Study Variables

Variable	1	2	3	4	5	6
1. Walking (%)	—	0.21**	0.19**	0.32***	0.27***	-0.41***
2. MVPA (%)	0.21**	—	0.07	0.03	0.01	-0.18**
3. Depression (%)	0.19**	0.07	—	0.39***	-0.04	0.12
4. Stress (%)	0.32***	0.03	0.39***	—	0.20**	-0.08
5. EQ-5D	0.27***	0.01	-0.04	0.20**	—	-0.44***
6. Aged 65+ (%)	-0.41***	-0.18**	0.12	-0.08	-0.44***	—

*p < 0.05, **p < 0.01, ***p < 0.001

MVPA showed no meaningful correlation with EQ-5D (*r* = 0.01, *p* = 0.84), although it demonstrated a weak positive association with walking participation (*r* = 0.21, *p* < 0.01). Depression and stress were positively correlated with each other (*r* = 0.39, *p* < 0.001). While depression showed no significant correlation with EQ-5D (*r* = -0.04, *p* = 0.58), stress demonstrated a positive correlation with EQ-5D (*r* = 0.20, *p* < 0.01).

The proportion of adults aged 65 years and older was negatively correlated with both walking participation (*r* = -0.41, *p* < 0.001) and EQ-5D (*r* = -0.44, *p* < 0.001), suggesting that district-level aging structure may account for part of the observed ecological associations between walking participation and HRQoL.

### Regression models association with HRQoL

Table 3 summarizes the regression analyses examining ecological associations between physical activity, mental health indicators, and HRQoL.

**Table 3:** Multiple Regression Models Examining Associations with EQ-5D

Predictor	Model 1β (SE)	Model 2β (SE)	Model 3β (SE)	Model 4β (SE)
Walking (%)	0.000440 (0.000101)***	—	0.000389 (0.000105)***	0.000107 (0.000110)
MVPA (%)	-0.000127 (0.000172)	—	-0.000120 (0.000169)	-0.000010 (0.000160)
Depression (%)	—	-0.001168 (0.000570)*	-0.001279 (0.000557)*	-0.001028 (0.000525)†
Stress (%)	—	0.001264 (0.000342)***	0.000916 (0.000347)**	0.000122 (0.000355)
Aged 65+ (%)	—	—	—	-0.000788 (0.000140)***
R²	0.077	0.059	0.113	0.223
Adjusted R²	0.069	0.050	0.097	0.206
N	229	229	229	229

Model 4 additionally adjusted for district-level aging structure using the proportion of adults aged 65 years and older

β unstandardized coefficient, *SE* standard error, *MVPA* moderate-to-vigorous physical activity

†*p* < 0.10, *p < 0.05, **p < 0.01, ***p < 0.001

#### Model 1: physical activity associations with HRQoL

Walking participation was significantly associated with higher EQ-5D scores (β = 0.000440, *p* < 0.001), whereas MVPA showed no significant association with HRQoL (*p* = 0.46). Model 1 explained 7.7% of the variance in HRQoL (R² = 0.077).

#### Model 2: mental health associations with HRQoL

Depression was negatively associated with EQ-5D (β = -0.001168, *p* < 0.05), whereas stress demonstrated a positive association with EQ-5D (β = 0.001264, *p* < 0.001). Model 2 explained 5.9% of the variance in HRQoL (R² = 0.059).

#### Model 3: full model

In the full model including all predictors, walking participation remained significantly associated with higher HRQoL (β = 0.000389, *p* < 0.001). Depression was negatively associated with HRQoL (β = -0.001279, *p* < 0.05), whereas stress remained positively associated with HRQoL (β = 0.000916, *p* < 0.01). MVPA remained non-significant. The full model explained 11.3% of the variance in HRQoL (R² = 0.113).

#### Model 4: aging-adjusted model

Model 4 additionally adjusted for district-level aging structure using the proportion of adults aged 65 years and older. After adjustment, the association between walking participation and HRQoL was attenuated and was no longer statistically significant (β = 0.000107, *p* = 0.332). Stress was also attenuated and became non-significant (β = 0.000122, *p* = 0.731). In contrast, the proportion of adults aged 65 years and older showed a significant negative association with HRQoL (β = -0.000788, *p* < 0.001). The explanatory power of the model increased substantially from R² = 0.113 in Model 3 to R² = 0.223 in Model 4, indicating that district-level aging structure accounted for a meaningful proportion of regional variation in HRQoL.

### Additional ecological analyses of walking participation and mental health indicators

To explore potential ecological relationships, additional regression analyses examined whether walking participation was positively associated with district-level depression and stress.

Walking participation was significantly associated with higher depressive symptoms (β = 0.0338, *p* = 0.007) and higher perceived stress (β = 0.1013, *p* < 0.001).

These findings suggest that district-level ecological associations between walking participation and mental health indicators may differ from individual-level relationships. The results are presented in Table 4.

**Table 4:** Ecological Associations Between Walking Participation and Mental Health Indicators

Outcome Variable	β	SE	*p*-value
Depression (%)	0.0338	0.0124	0.007
Stress (%)	0.1013	0.0200	< 0.001

Additional sensitivity analyses adjusting for district-level aging structure yielded broadly similar directions of association. However, the association between walking participation and HRQoL was attenuated after adjustment for the proportion of older adults aged 65 years and above. District-level aging structure itself showed a significant negative association with HRQoL, suggesting that demographic composition may partially account for regional variation in HRQoL across districts.

## Discussion

Several notable findings emerged. First, walking participation showed positive ecological associations with HRQoL in the initial models; however, these associations were substantially attenuated and became non-significant after adjustment for district-level aging structure. At the same time, model explanatory power increased considerably following inclusion of the proportion of adults aged 65 years and older. These findings suggest that demographic composition may account for an important part of the initially observed ecological association between walking participation and HRQoL across districts. These findings are broadly consistent with prior individual-level research suggesting that walking may support physical function, psychological well-being, and overall quality of life [1–6, 10–12, 24–28]. In contrast, MVPA showed no meaningful association in our models, suggesting that at the district level, moderate-to-vigorous activity may not capture community-wide differences in quality of life as effectively as walking does. These findings reinforce the importance of considering demographic composition when interpreting ecological associations between walking participation and HRQoL across districts.

One of the most notable findings was the positive association between perceived stress and HRQoL at the district level. This pattern differs from most individual-level studies, which generally report negative associations between stress and quality of life [16–18]. Importantly, the present finding should not be interpreted as indicating that stress is beneficial for HRQoL at the individual level. Rather, the association may reflect broader contextual or compositional differences across districts. For example, some districts may simultaneously report higher average stress levels and higher average HRQoL due to broader compositional or contextual differences that were not directly measured in the present study. These interpretations should therefore be regarded as hypothetical ecological explanations rather than established contextual mechanisms. These findings therefore highlight the importance of cautious interpretation in ecological analyses, where population-level associations may differ substantially from individual-level relationships [19, 20, 29–33].

These discrepancies reinforce the need to avoid assuming that individual- and group-level relationships will necessarily align. The divergence resembles classic issues raised in discussions of ecological fallacy, where associations observed at the population level may not hold for individuals [13, 14, 24, 34–38]. The findings should also be interpreted in light of both ecological and atomistic fallacies. Ecological associations observed at the district level cannot be assumed to apply directly to individuals, just as individual-level findings may not necessarily explain population-level regional patterns. Accordingly, the present results should be understood as reflecting district-level population associations rather than causal mechanisms operating at the individual level. Unmeasured district-level compositional or contextual characteristics may potentially contribute to these ecological associations, although these variables were not directly included in the present analyses [21–23, 25–27, 39, 40]. Future studies incorporating more district-level covariates—such as income, walkability indices, or environmental conditions—may help explain why mental health indicators behave differently across communities [27, 29–31].

This study benefits from the use of comprehensive nationwide data, enhancing the robustness and generalizability of its ecological findings [21–23]. It also contributes to the literature by exploring physical activity and HRQoL at a community level, where district-level ecological associations are particularly relevant. Several limitations should also be acknowledged. Ecological analyses cannot establish causal relationships or be generalized to individual outcomes [13, 14, 35–38]. In addition, the present ecological design does not allow compositional explanations (differences in the characteristics of residents across districts) to be clearly separated from broader contextual explanations related to district-level environments or regional structures. Although district-level aging structure was included in additional analyses, other potentially important contextual factors such as socioeconomic status, urbanization, healthcare access, social capital, neighborhood safety, and built-environment characteristics were not available in the present dataset and may also have contributed to the observed ecological associations. EQ-5D scores in Korea also tend to cluster at the higher end of the scale, which may limit variability and reduce detectable effect sizes [7–9, 34]. In addition, the EQ-5D scores demonstrated relatively limited variability across districts, reflecting a potential ceiling effect commonly observed in population-based HRQoL measures in Korea. This restricted variance may partially explain the relatively modest R² values observed in the regression models. This limited variance may have constrained detectable effect sizes and reduced overall model explanatory power.

Overall, the findings suggest that walking participation may represent a potentially relevant population-level marker associated with regional HRQoL patterns, although mental health indicators appeared to function differently at broader ecological scales and the present ecological analyses do not establish that increasing walking alone would directly improve district-level HRQoL. Future public health research may benefit from considering how district-level demographic and contextual characteristics relate to ecological patterns in physical activity, mental health, and HRQoL [21–23, 25–33, 39, 40]. Ecological perspectives can therefore complement individual-level evidence by identifying how community-level characteristics may be associated with broader population health patterns [35–40].

## Conclusions

This ecological analysis of 229 Korean districts found that walking participation was associated with HRQoL in the initial models; however, these associations were substantially attenuated after adjustment for district-level aging structure. The findings further suggest that demographic composition may account for an important proportion of regional variation in HRQoL across districts. Walking participation may nevertheless represent a potentially relevant population-level indicator associated with regional HRQoL patterns, although these ecological findings do not establish that increasing walking alone would directly improve district-level HRQoL [1–6, 10–12, 25–28].

At the same time, the study reveals complex and context-dependent relationships between mental health and HRQoL. Higher levels of depressive symptoms were associated with lower HRQoL, whereas perceived stress showed a positive association at the ecological level— highlighting the possibility that district-level ecological associations may differ from individual-level mental health relationships [30–33, 35–40]. These findings further highlight the importance of cautious interpretation when examining ecological associations between mental health indicators and HRQoL across districts.

Overall, this study contributes to a growing body of ecological research emphasizing that population-level health patterns cannot be inferred solely from individual-level findings [13, 14, 35–38]. Future research should incorporate additional district-level covariates—including walkability, economic indicators, and social determinants—to elucidate ecological relationships more clearly [25–33, 39, 40]. Future research should further investigate how district-level demographic and contextual characteristics relate to ecological patterns in HRQoL and mental health indicators across regions.

## Data Availability

All aggregated district-level datasets used in this study are publicly available through the Korean Statistical Information Service (KOSIS) ( [https://kosis.kr](https:/kosis.kr) ). No individual-level or identifiable data were accessed.

## Abbreviations

HRQoL: Health-related quality of life
PA: Physical activity
MVPA: Moderate-to-vigorous physical activity
EQ-5D: EuroQol five-dimension scale
KOSIS: Korean statistical information service
IRB: Institutional review board
WHO: World Health Organization
